# A Type I Aggregation-Induced Emission Photosensitizer Enables Peroxide-Free Photodynamic Tooth Whitening While Preserving Enamel Integrity

**DOI:** 10.3390/jfb17090466

**Published:** 2026-09-10

**Authors:** Kaiqi Peng, Jingheng Liang, Yiyi Huang, Yixue Li, Feng-Shou Liu, Yan Zhou

**Affiliations:** 1 Hospital of Stomatology, Guanghua School of Stomatology, Sun Yat-sen University, Guangzhou 510000, China; pengkq5@mail2.sysu.edu.cn (K.P.); liangjh228@mail.sysu.edu.cn (J.L.); huangyiyi99@mail2.sysu.edu.cn (Y.H.); liyx529@alumni.sysu.edu.cn (Y.L.); 2 School of Chemistry and Chemical Engineering, Guangdong Pharmaceutical University, Zhongshan 528400, China

**Keywords:** aggregation-induced emission, photodynamic therapy, tooth whitening, enamel safety

## Abstract

Conventional hydrogen peroxide (HP) tooth whitening treatments frequently induce structural deterioration of enamel, necessitating the development of biocompatible and highly efficient alternatives. This in vitro study investigated the application of a novel functional biomaterial, the Type I aggregation-induced emission (AIE) photosensitizer CPTQ, for peroxide-free photodynamic tooth whitening. Under white-light irradiation (25 mW/cm^2^), CPTQ (12.5 μM) demonstrated rapid degradation of representative chromogenic molecules (crystal violet, malachite green, and rhodamine B). Extracted human teeth stained with both model pigments and complex beverage mixtures (coffee, tea, and fruit juices) were allocated into four treatment groups: negative control (NC), CPTQ, 7.5% HP, and 30% HP (*n* = 6 per group). Colorimetric parameters (Δ*E*_00_, Δa*, Δb*, and ΔL*) and enamel-related endpoints—including surface morphology, roughness, mineral composition (Ca/P), and microhardness—were evaluated over 6 h and analyzed using one-way ANOVA. For pigment-stained teeth, the photodynamic whitening efficiency of CPTQ was comparable to that of 30% HP (Δ*E*_00_, *p* > 0.05) during the 6 h treatment period. At 1.5 h, the Δ*E*_00_ value in the CPTQ group (23.35 ± 2.32) was significantly greater than those in the NC group (15.70 ± 1.78, *p* < 0.05) and the 7.5% HP group (15.02 ± 1.63, *p* < 0.05). For beverage-stained teeth, the whitening efficiency (Δ*E*_00_) of CPTQ was significantly greater than that of the control group at 1.5 h (6.22 ± 2.09 vs. 1.82 ± 0.56, *p* < 0.01) and was significantly greater than that of 7.5% HP at 4.5 h (10.94 ± 2.82 vs. 7.56 ± 1.78, *p* < 0.05). Crucially, unlike the change observed after 30% HP treatments, CPTQ treatment resulted in no statistically significant differences from NC in preserved enamel surface integrity, including morphology assessed by SEM, surface roughness (*R*_a_ and *S*_a_, both *p* > 0.05), mineral composition (Ca and P, both *p* > 0.05), and microhardness (Δ*H*_V_, *p* > 0.05). Mechanistic investigations using reactive oxygen species (ROS) scavengers (TBA and DABCO) suggested that hydroxyl radicals generated via the Type I photodynamic pathway are the primary drivers of pigment degradation, reducing Δ*E*_00_ from 12.56 to 1.09 upon •OH inhibition. CPTQ achieved measurable in vitro whitening through a predominantly Type I ROS-mediated mechanism while causing limited changes in the evaluated enamel surface endpoints.

## 1. Introduction

With the improvement of living standards, public concern has extended beyond oral disease prevention to dental aesthetics. Epidemiological surveys indicate that 48.9% of the Chinese population experience tooth discoloration, and 52.6% are dissatisfied with their tooth color [1]. Similarly, 34% of adults in the United States [2] and approximately half of the population in the United Kingdom report concerns regarding stained teeth [3]. These data highlight the increasing demand for effective and safe tooth whitening strategies.

Exogenous staining is a common cause of tooth discoloration, which may result from inadequate oral hygiene, smoking, and consumption of pigmented foods such as tea and coffee, as well as exposure to certain medications like chlorhexidine [4,5,6,7]. According to the widely accepted chromophore theory, dental stains are mainly associated with organic molecules containing conjugated single and double bonds, often together with heteroatoms, carbonyl groups, and aromatic rings [8,9,10]. Tooth whitening fundamentally relies on the oxidative breakdown of these conjugated structures by reactive oxygen species (ROS), possibly converting chromophoric molecules into smaller and less colored compounds [11]. At present, hydrogen peroxide (HP) and carbamide peroxide (CP) are the most commonly used tooth whitening agents in clinical practice. HP exerts its whitening effect through the generation of reactive oxygen species (ROS), which oxidize and degrade chromogenic molecules [12]. However, the application of HP has certain limitations. Due to its favorable permeability, HP can diffuse through enamel and dentin into the pulp chamber, potentially triggering pulp inflammation and sensitivity [13,14]. Its oxidative and cytotoxic properties may also activate inflammatory pathways, including the TNF-α pathway in surrounding tissues [15]. Moreover, peroxide treatment has been reported to alter enamel surface properties, including reduced microhardness and increased surface roughness, which may favor pigment redeposition and bacterial adhesion [16,17], thereby posing potential threats to both oral and even overall systemic health [18]. Therefore, tooth whitening approaches based on more controllable ROS generation remain worthy of investigation, particularly with regard to balancing whitening effect and enamel surface preservation.

Photodynamic therapy (PDT) provides a light-triggered approach for ROS generation. PDT utilizes photosensitizers activated by a specific light source to generate ROS and has emerged as a promising approach for controllable therapeutic applications such as antimicrobial treatment and anticancer therapy [19,20,21]. Notably, certain ROS generated during PDT possess higher oxidation potentials than H_2_O_2_—for example, •OH (2.7 V) and ^1^O_2_ (2.2 V)—both exceeding that of H_2_O_2_ (1.77 V) [22]. Higher oxidation potentials enable more effective cleavage of conjugated chromophores [23], potentially achieving effective whitening at a lower overall oxidative burden [24,25]. Previous studies have reported that PDT-based tooth whitening showed minimal structural alteration to dental hard tissues [26,27,28], suggesting improved safety compared to high-concentration peroxide systems.

**Figure 1:**
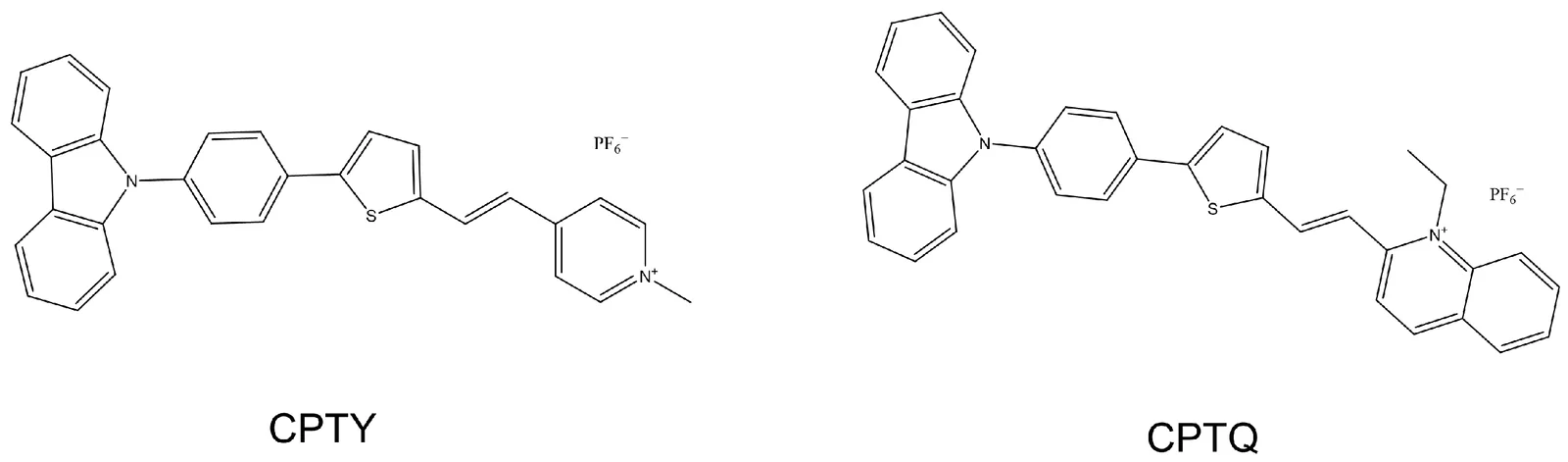
Chemical structures of CPTY and CPTQ.

The development of high-performance photosensitizers is central to the advancement of PDT-based whitening. Aggregation-induced emission luminogens (AIEgens) are a class of photosensitizers that overcome the aggregation-caused quenching (ACQ) commonly observed in conventional systems [29,30]. Unlike traditional photosensitizers, AIEgens exhibit enhanced fluorescence and elevated ROS generation efficiency in aggregated states, making them suitable for applications in heterogeneous biological environments [31]. In our previous studies, we reported a series of AIEgens, including CPTY and CPTQ (Figure 1), with Type I ROS generation and antibacterial photodynamic activity [32,33]. Given their Type I ROS-generating ability, we hypothesized that these AIEgens may exhibit significant photodynamic whitening capacity toward extrinsic dental pigments.

Although photodynamic approaches have been investigated for tooth whitening, their whitening effect and influence on enamel surfaces have not been fully clarified. In this study, we examined AIEgen-mediated photodynamic whitening in vitro. We first compared the ability of AIEgens and hydrogen peroxide to degrade representative chromogenic molecules. CPTQ was then selected for further evaluation in extracted human teeth stained with either model pigments or a beverage mixture. Enamel surface morphology, roughness, calcium/phosphorus composition, and microhardness were assessed to determine whether the treatment caused detectable surface changes. ROS scavenger experiments were also performed to explore the possible roles of Type I and Type II ROS in pigment degradation and tooth whitening. This study aimed to determine whether CPTQ-mediated photodynamic treatment could produce measurable whitening effects while causing limited changes in the evaluated enamel surface parameters. We hypothesized that CPTQ-mediated photodynamic treatment would achieve greater tooth whitening than the light-only control while causing fewer changes in the evaluated enamel parameters than 30% HP.

## 2. Materials and Methods

### 2.1. Photodecomposition of Model Pigments by AIEgens

To evaluate the photodynamic decomposition efficacy, three coloring molecules commonly used in previous studies [27,34], namely crystal violet (CV, Macklin, Shanghai, China), malachite green (MG, Macklin, Shanghai, China), and rhodamine B (RhB, Macklin, Shanghai, China), were employed as model dyes, and the pigments were prepared at a concentration of 25 μM. The photosensitizers CPTQ and CPTY were added at concentrations of 3.125, 6.25, 12.5, and 25 μM, corresponding to photosensitizer-to-pigment molar ratios of 1:8, 1:4, 1:2, and 1:1, respectively. Based on the optimal illumination conditions established for CPTY and CPTQ, the mixed solution was irradiated using a white light source at an irradiance of 25 mW/cm^2^ [32,33]. The decomposition process was monitored by recording the absorbance of the pigments at 15 min intervals over a total period of 90 min. The concentration of each pigment was determined using a microplate reader (BioTek Epoch 2, Winooski, VT, USA) at specific wavelengths: 584 nm (CV), 618 nm (MG), and 554 nm (RhB). Pigment concentrations at each time point were calculated based on absorbance values, and degradation kinetics were plotted as concentration–time curves. Three independent experiments were performed.

### 2.2. Evaluation of Pigment Decomposition Efficiency Between AIEgens and HP

To compare the decomposition efficacy of various treatments on model pigments, five experimental groups were established: Negative Control, 7.5% HP (Aladdin, Shanghai, China), 30% HP (Aladdin, Shanghai, China), 12.5 μM CPTY, and 12.5 μM CPTQ. The decomposition effects were assessed on three model pigments—CV, MG, and RhB—using the respective treatment conditions. For the CPTY and CPTQ treatment groups, the solutions were irradiated with a white light source at a power density of 25 mW/cm^2^. All treatments were applied for 90 min. Pigment concentrations before and after treatment were determined using a microplate reader at 584 nm (CV), 618 nm (MG), and 554 nm (RhB). Decomposition rates were calculated and statistically compared among groups. Three independent experiments were performed.

### 2.3. Tooth Collection and Whitening Protocol for Classic Model Pigment-Stained Extracted Teeth

Extracted human third molars were collected from donors who provided written informed consent, following approval by the Ethics Committee of the Hospital of Stomatology, Sun Yat-sen University (No. KQEC-2026-008-01). The age range of the sourced population was 18 to 30 years. Only intact teeth were selected. Teeth with caries, fluorosis, tetracycline staining, or enamel hypoplasia were excluded. After removing periodontal ligaments, calculus, and debris, the teeth were ultrasonically cleaned for 20 min and stored in PBS solution. In this experiment, 24 extracted teeth were stained by immersion in a mixed solution containing 25 μM each of CV, MG, and RhB for 14 days with a tooth-to-solution volume ratio of 1:10 (mL). Following staining, the teeth were rinsed with distilled water for 30 min to remove loosely bound pigment. The specimens were then randomly divided into four groups (*n* = 6 per group): Negative Control (NC), 7.5% HP, 30% HP, and CPTQ. CPTQ was first dissolved in DMSO (1 mM) and diluted with PBS to 12.5 μM. Extracted teeth were immersed in 5 mL of the CPTQ solution and continuously irradiated for 6 h with white light at an irradiance of 25 mW/cm^2^ and a source-to-enamel distance of 20 cm; the solution was renewed every 1.5 h. The NC group received the identical vehicle without CPTQ and the same irradiation protocol. The whitening agent was refreshed every 1.5 h. Photographs were taken at 0.5 h, 1.5 h, 3 h, 4.5 h, and 6 h, and a miniature photographic studio was used to ensure the reproducibility of the imaging conditions. Since the blue pigmentation of the tooth specimens resulted in reading failures by the VITA Easyshade V spectrophotometer (VITA Zahnfabrik, Bad Säckingen, Germany), image processing software (Adobe Photoshop 26.0) was used instead to extract the color parameters in the CIELAB color space (L*, a*, b*). Image acquisition was performed in a miniature photographic studio under consistent lighting and camera configurations. Color change (Δ*E*_00_), as well as changes in lightness (ΔL*), red–green coordinate (Δa*), and yellow–blue coordinate (Δb*), were calculated and compared according to the CIEDE2000 color difference formula [35]:

$$\Delta E_{00}=\sqrt{{\left(\frac{\Delta L^{\prime }}{k_{L}S_{L}}\right)}^{2}+{\left(\frac{\Delta C^{\prime }}{k_{C}S_{C}}\right)}^{2}+{\left(\frac{\Delta H^{\prime }}{k_{H}S_{H}}\right)}^{2}+R_{T}\left(\frac{\Delta C^{\prime }}{k_{C}S_{C}}\right)\left(\frac{\Delta H^{\prime }}{k_{H}S_{H}}\right)}$$

### 2.4. Whitening Protocol for Beverage-Stained Extracted Teeth

In this experiment, 24 extracted teeth were stained by immersion in a mixed beverage solution containing coffee, tea, blueberry juice, and grape juice at a volume ratio of 1:1:1:1 with a tooth-to-solution volume ratio of 1:10 (mL). The staining process was carried out for 14 days. After staining, the teeth were rinsed with distilled water for 30 min to remove any superficially adsorbed pigments. The specimens were then randomly divided into four groups (*n* = 6 per group): negative control, 7.5% HP, 30% HP, and CPTQ. The whitening procedure was conducted over 6 h, with the whitening agent refreshed every 1.5 h. Standardized photographs and color measurements were taken at 0.5, 1.5, 3, 4.5, and 6 h. A miniature photographic studio was used to ensure the reproducibility of the imaging conditions. Parameters in the CIELAB color space (L*, a*, b*) were measured using VITA Easyshade V (VITA Zahnfabrik, Bad Säckingen, Germany). Color change (Δ*E*_00_), as well as changes in lightness (ΔL*), red–green coordinate (Δa*), and yellow–blue coordinate (Δb*), were calculated and compared according to the CIEDE2000 color difference formula [35].

### 2.5. Identification of Reactive Oxygen Species (ROS) Involved in Pigment Decomposition by CPTQ

To investigate the roles of different ROS in the photodynamic decomposition of model pigments by CPTQ, specific ROS scavengers were introduced into the reaction system. Hydroxyl radicals (•OH) and singlet oxygen (^1^O_2_) were scavenged using tert-butyl alcohol (TBA, Macklin, Shanghai, China) and 1,4-diazabicyclo[2.2.2]octane (DABCO, Macklin, Shanghai, China), respectively. TBA (40%, *v*/*v*) and DABCO (5 mM) were separately added to the CPTQ solution (12.5 μM) containing each model pigment—CV, MG, and RhB. The mixtures were then irradiated with a white light source at a power density of 25 mW/cm^2^. After treatment, the residual pigment concentrations were measured using a microplate reader at the specific wavelengths of 584 nm (CV), 618 nm (MG), and 554 nm (RhB). The normalized decomposition rates were calculated for each condition to evaluate the contribution of each type of ROS to the overall pigment decomposition. Three independent experiments were performed.

### 2.6. Identification of Reactive Oxygen Species (ROS) Involved in Tooth Whitening by CPTQ

To further elucidate the roles of specific reactive oxygen species (ROS) in the CPTQ-mediated whitening process on beverage-stained teeth, ROS inhibition experiments were conducted using tert-butyl alcohol (TBA) and 1,4-diazabicyclo[2.2.2]octane (DABCO) as scavengers for hydroxyl radicals and singlet oxygen (^1^O_2_), respectively. In this experiment, 18 extracted teeth were first stained by immersion in a mixed beverage solution containing coffee, tea, blueberry juice, and grape juice at a volume ratio of 1:1:1:1 for 14 days. The stained teeth (*n* = 6 per group) were then subjected to whitening treatments with CPTQ (12.5 μM) under irradiation at 25 mW/cm^2^, in the presence or absence of DABCO (5 mM) or TBA (40%, *v*/*v*). Whitening was conducted over 6 h with solution replacement every 1.5 h. At designated time points (0.5, 1.5, 3, 4.5, and 6 h), photographs were taken, and color parameters in the CIELab color space were recorded using VITA Easyshade V (VITA Zahnfabrik, Bad Säckingen, Germany). Color differences (Δ*E*_00_) were calculated according to the CIEDE2000 color difference formula [35] and compared among groups to evaluate the contribution of each ROS type to the overall whitening effect.

### 2.7. Enamel Structure Evaluation

The effects of different treatments on enamel structure were evaluated using multiple surface analysis techniques. Extracted human teeth were treated with PBS (negative control group), CPTQ, and 30% HP under the conditions described above. After treatment, the surface morphology of the enamel was examined using field emission scanning electron microscopy (FE-SEM, Apreo 2 S, Thermo Fisher Scientific, Brno, Czech Republic) (*n* = 3 per group), and the relative contents of calcium (Ca) and phosphorus (P) on the enamel surface were simultaneously determined by energy-dispersive X-ray spectroscopy (EDS) attached to the SEM. Surface roughness was assessed using a laser confocal microscope (Olympus OLS4100, Tokyo, Japan). Both roughness average (*R*_a_) and surface roughness average (*S*_a_) were measured and calculated for specimens from each treatment group (*n* = 6 per group). Additionally, the Vickers hardness of the enamel surface was measured using a microhardness tester (HMV-2T SHIMADZU, Kyoto, Japan) to evaluate changes in mechanical properties following different treatments *(n* = 6 per group). The paired changes (Δ*H*_V_) at baseline and post-treatment were calculated and compared among groups.

### 2.8. Statistical Analysis

Results are presented as the mean ± standard deviation (SD). Statistical analysis was performed using SPSS 26.0. First, the normality (Shapiro–Wilk test) and homogeneity of variance (Levene’s test) of the data were verified. As the data involved multiple groups, one-way ANOVA was used to compare differences among groups. When the one-way ANOVA results showed statistically significant differences among groups (*p* < 0.05), pairwise comparisons were conducted using a post hoc test (Tukey‘s HSD).

## 3. Results

### 3.1. Degradation of Pigments by AIEgens

**Figure 2:**
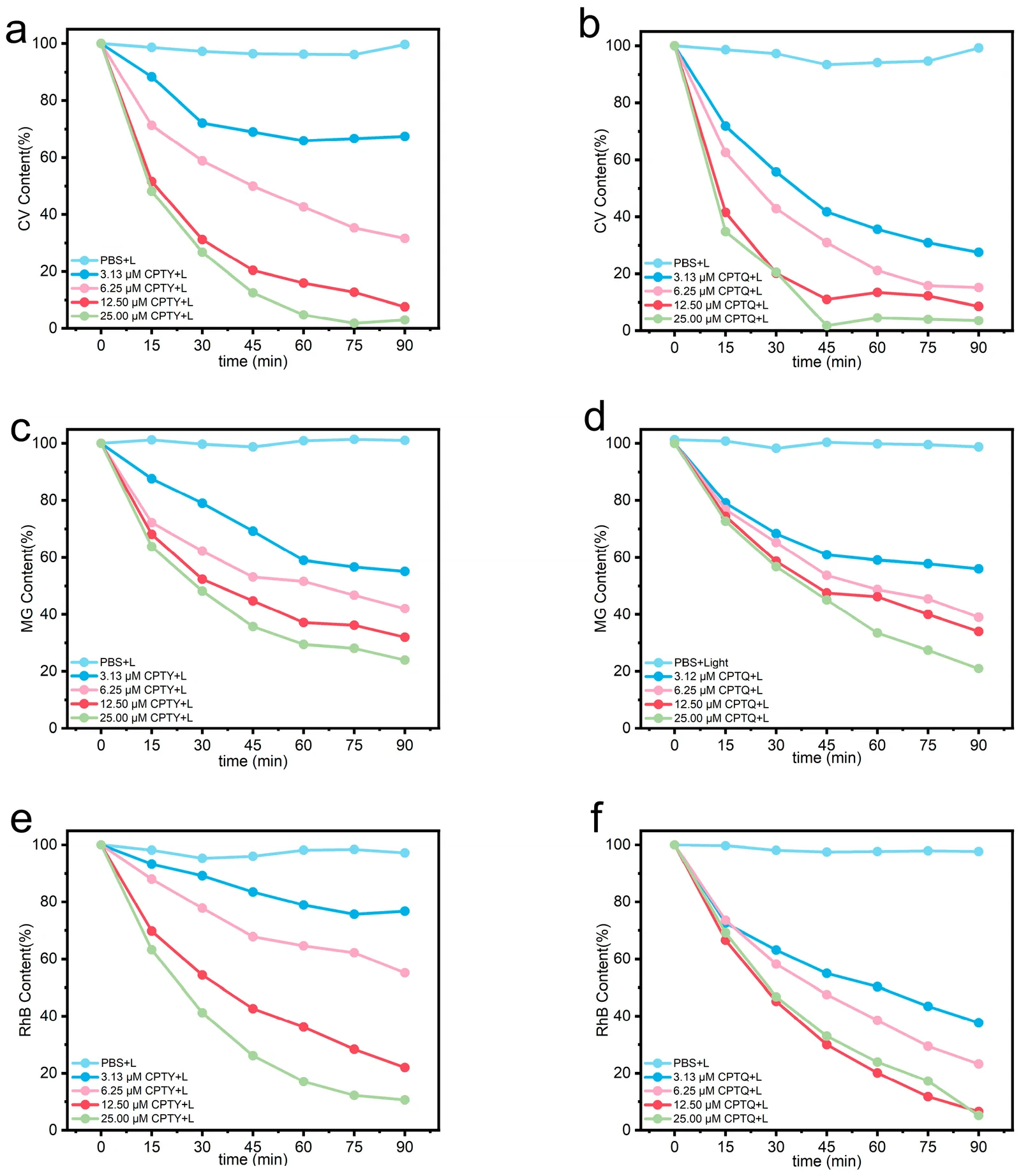
Degradation of CV, MG and RhB by CPTY and CPTQ. Photodegradation profiles of CV (**a**), MG (**c**), and RhB (**e**) treated with four concentrations of CPTY. Photodegradation profiles of CV (**b**), MG (**d**), and RhB (**f**) treated with four concentrations of CPTQ.

To preliminarily investigate the whitening capability of AIEgens, two previously reported AIEgens from our research group, CPTY and CPTQ, were selected. The degradation of three representative organic pigments—crystal violet (CV), malachite green (MG), and rhodamine B (RhB)—by the AIEgens was evaluated. Pigment concentrations were monitored using a microplate reader at 15 min intervals over a 90 min period. Time–concentration curves were generated accordingly. Under light irradiation, both CPTQ and CPTY exhibited time- and concentration-dependent dye degradation, with greater reductions observed at longer irradiation times and higher AIEgen concentrations. After 90 min of irradiation, CPTY at concentrations of 3.125 μM, 6.25 μM, 12.5 μM, and 25 μM reduced CV concentration to 67.41%, 31.67%, 7.64%, and 2.91% (Figure 2a); reduced MG concentration to 55.12%, 42.27%, 32.9%, and 24.28% (Figure 2c); and reduced RhB concentration to 76.79%, 55.16%, 22.03%, and 10.67% (Figure 2e). Under the same 90 min of irradiation, treatment with CPTQ at concentrations of 3.125 μM, 6.25 μM, 12.5 μM, and 25 μM reduced CV concentration to 27.59%, 15.10%, 8.53%, and 3.25% (Figure 2b); reduced MG concentration to 61.68%, 38.49%, 34.46%, and 21.87% (Figure 2d); and reduced RhB concentration to 37.73%, 23.25%, 6.50%, and 5.18% (Figure 2f). The degradation curves for CV plateaued at 45–60 min, whereas MG and RhB reached stabilization at about 90 min. Considering both reaction completeness and efficiency, 90 min of irradiation was selected for subsequent experiments. Based on the degradation kinetics, 90 min was selected as the irradiation time to ensure a sufficient reaction between the AIEgens and the pigment. A clear dose–response relationship was observed, with marked enhancement in pigment decomposition as the concentration increased from 3.125 μM to 6.25 μM and further to 12.5 μM. However, doubling the concentration from 12.5 μM to 25 μM resulted in only marginal additional reduction, indicating a tendency toward saturation. Accordingly, 90 min of irradiation and 12.5 μM AIEgens were chosen as the standard conditions for further comparison with HP.

### 3.2. Pigment-Degrading Efficiency: AIEgens Compared to Hydrogen Peroxide

**Figure 3:**
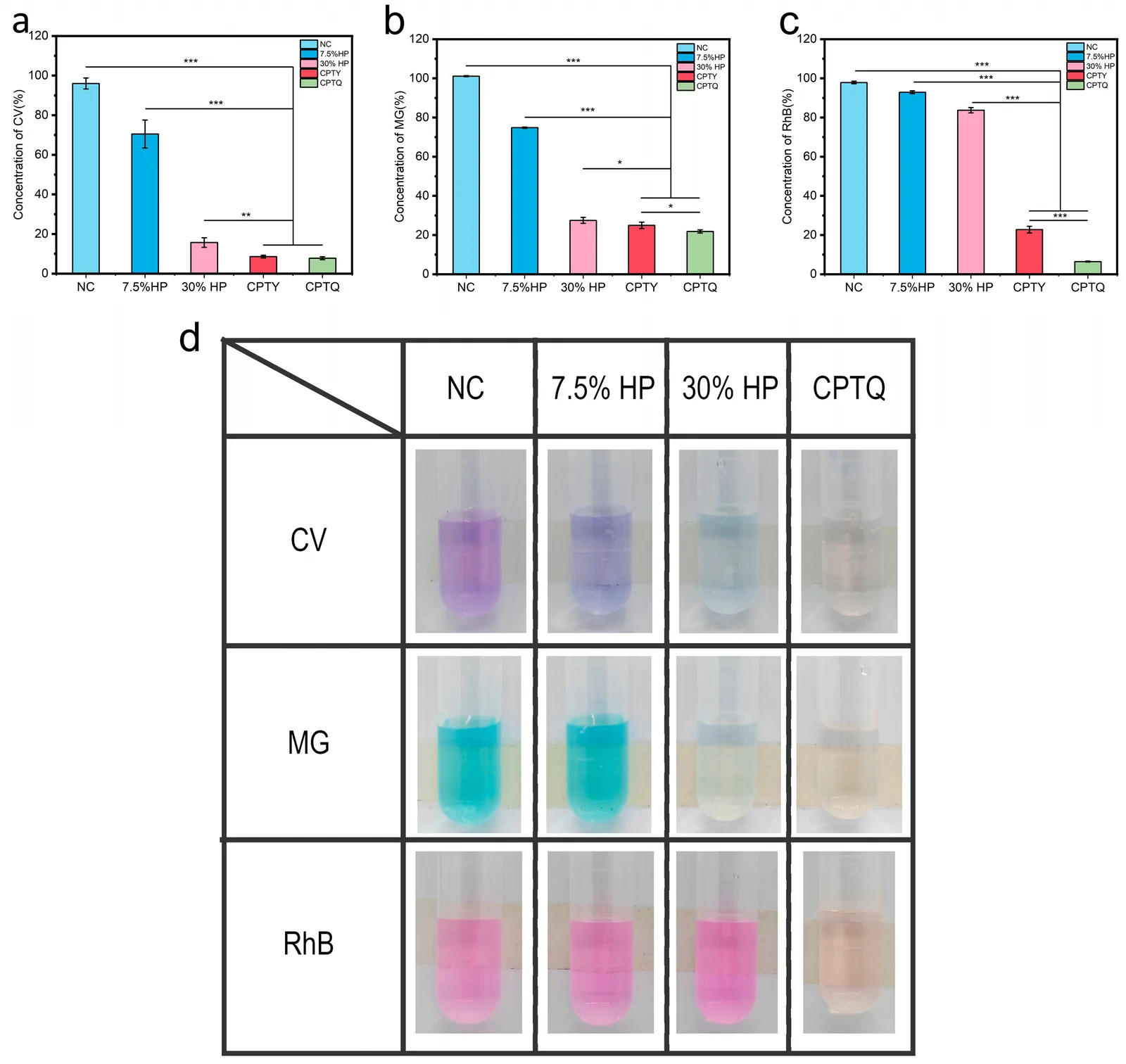
Comparison of pigment degradation rates between AIEgens and HP. Degradation rates of CV (**a**), MG (**b**), and RhB (**c**) after 90 min of treatment. (**d**) Representative photographs of three pigment solutions after 90 min exposure to CPTQ and HP. *** *p* < 0.001, ** *p* < 0.01, * *p* < 0.05.

Having established the pigment-degrading capability of AIEgens, we compared their effects with those of HP at 7.5% and 30%, representing home and in-office concentrations, respectively [8,36]. Pigment concentrations before and after 90 min treatment were quantified using a microplate reader, and degradation rates were calculated accordingly. The results revealed that the concentrations of CV after treatment with CPTY (8.59% ± 0.67%) and CPTQ (7.81% ± 0.75%) were significantly lower than those observed in the 7.5% HP (70.53% ± 7.06%, *p* < 0.001) and 30% HP (15.71% ± 2.4%, *p* < 0.01) (Figure 3a). Similarly, the concentrations of MG after treatment with CPTY (24.97% ± 1.61%) and CPTQ (21.83% ± 0.82%) were significantly lower than those in the 7.5% HP (74.82% ± 0.31%, *p* < 0.001) and in the 30% HP (27.50% ± 1.51%, *p* < 0.05) (Figure 3b). The concentrations of RhB after treatment with CPTY (22.80% ± 1.68%) and CPTQ (6.46% ± 0.22%) were significantly lower than those in the 7.5% HP (92.91% ± 0.74%, *p* < 0.001) and in the 30% HP group (83.74% ± 1.37%, *p* < 0.001) (Figure 3c). When comparing the two AIEgens, CPTQ exhibited superior degradation ability relative to CPTY for MG (*p* < 0.05, Figure 3b) and RhB (*p* < 0.001, Figure 3c). Consistent with the quantitative results, visual inspection of dye solutions after 90 min treatment showed markedly lighter coloration in the CPTQ group compared with the negative control group and the low- and high-concentration HP control groups (Figure 3d). Overall, CPTQ showed efficient degradation of the tested dyes and was therefore selected for subsequent tooth whitening experiments.

### 3.3. Whitening Effect of CPTQ on Extracted Teeth Stained with Model Pigment

**Figure 4:**
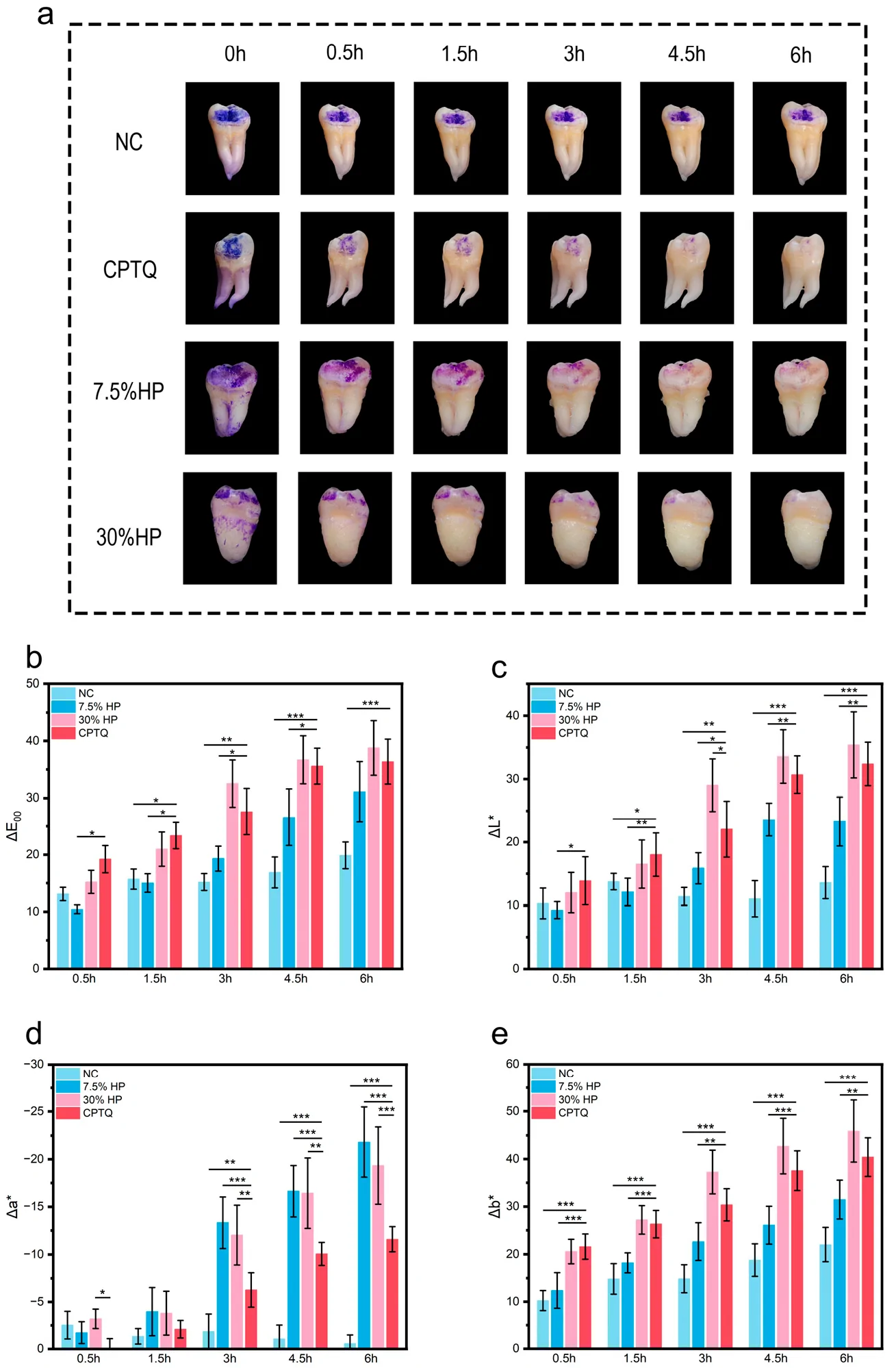
Whitening effect of CPTQ on extracted teeth stained with three model pigments: (**a**) Photographs showing the tooth color recorded by a camera at different time points. (**b**–**e**) Changes in Δ*E*_00_, ΔL*, Δa*, and Δb* for each group at different time points. *** *p* < 0.001, ** *p* < 0.01, * *p* < 0.05.

To evaluate the whitening efficacy of CPTQ on extracted teeth stained with pigment, we stained the teeth with an equal mixture of three model pigments for 14 days. The whitening treatment was performed for a total of 6 h, and color measurements were recorded at 0.5, 1.5, 3, 4.5, and 6 h. Overall, compared with the negative control group and the 7.5% HP treatment group, the CPTQ treatment group exhibited greater color change, while its effect was comparable to that of the 30% HP group under the present in vitro conditions. The photographs showed a marked reduction in visible staining in the CPTQ and 30% HP groups over time (Figure 4a). Analysis of color change (Δ*E*_00_) at the same time points (0.5 h, 1.5 h, 3 h, 4.5 h, and 6 h) revealed no significant difference in color change between the 30% HP group and the CPTQ group under the present in vitro conditions (*p* > 0.05, Figure 4b). Significant color change (Δ*E*_00_) was observed between the CPTQ group (23.35 ± 2.32) and both the control group (15.70 ± 1.78) and the 7.5% HP group (15.02 ± 1.63) after just 1.5 h of treatment (both *p* < 0.05, Figure 4b), indicating its rapid whitening effect on extrinsically stained teeth in vitro. Changes in lightness (ΔL*) further supported these findings. The L* value increased during CPTQ treatment (Figure 4c), indicating enhanced brightness of the pigment-stained teeth. A significant ΔL* difference between CPTQ (18.03 ± 3.43) and the control group (13.77 ± 1.29) was reached at 1.5 h (*p* < 0.05, Figure 4c). For the red–green coordinate, the a* value decreased over time (Figure 4d), indicating a reduction in tooth redness during the CPTQ whitening process. A significant difference in Δa* was observed between CPTQ (−6.27 ± 1.83) and the control group (−1.86 ± 1.84) at 3 h (*p* < 0.01, Figure 4d). For changes in the yellow–blue coordinate, the b* value increased over time (Figure 4e), indicating a reduction in tooth blueness during the CPTQ whitening process. The CPTQ group (21.63 ± 2.63) showed a significant difference from the control group (10.23 ± 2.13) as early as 0.5 h (*p* < 0.001, Figure 4e).

### 3.4. Whitening Effect of CPTQ on Beverage-Stained Extracted Teeth

**Figure 5:**
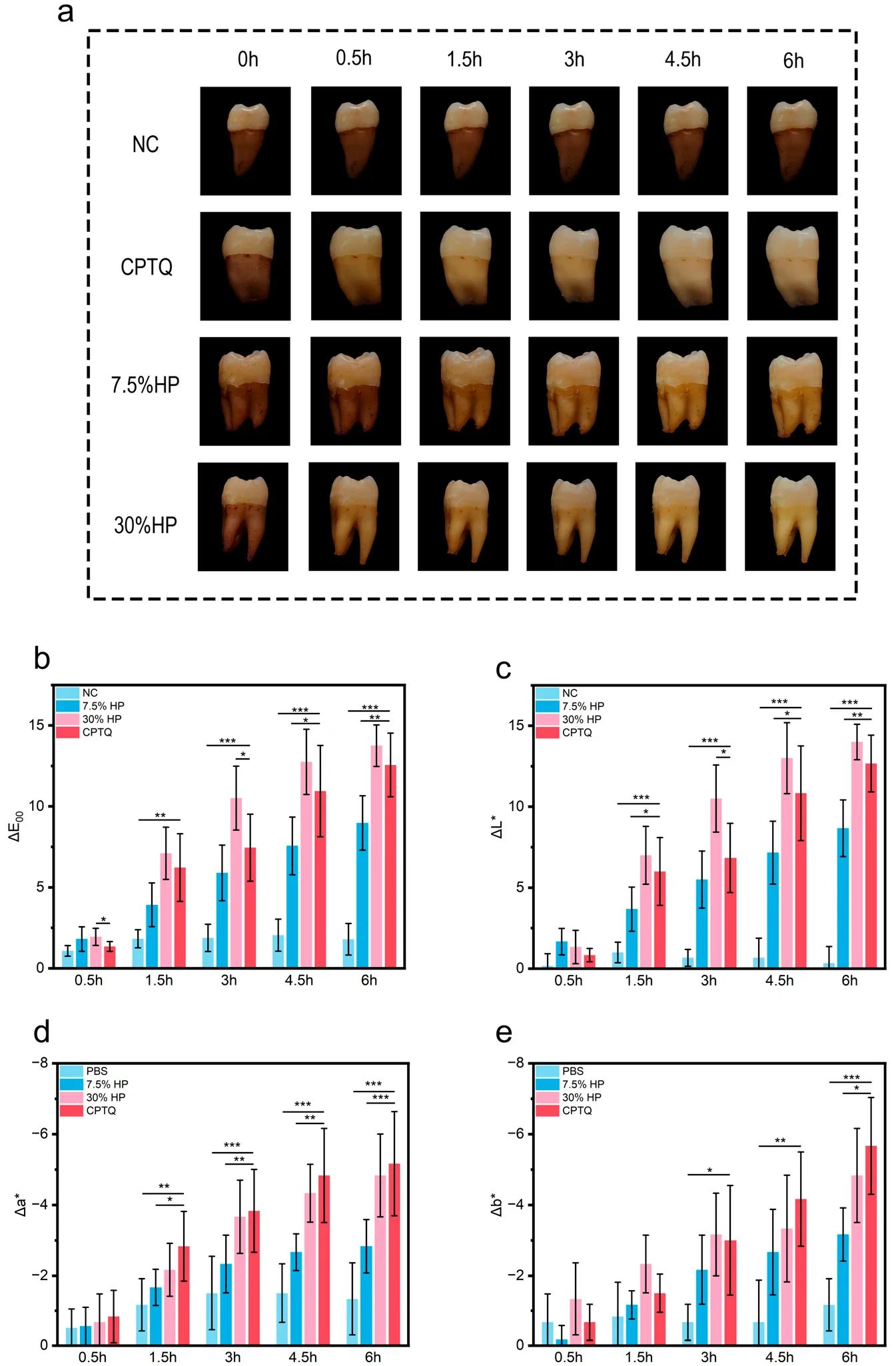
Whitening effect of CPTQ on extracted teeth stained with beverage: (**a**) Photographs showing the tooth color recorded by a camera at different time points. (**b**–**e**) Changes in Δ*E*_00_, ΔL*, Δa*, and Δb* for each group at different time points. *** *p* < 0.001, ** *p* < 0.01, * *p* < 0.05.

To better simulate clinically relevant staining conditions, extracted teeth were immersed for 14 days in a mixture of coffee, tea, blueberry juice, and grape juice (mixed in a 1:1:1:1 volume ratio). The whitening efficacy of CPTQ was then evaluated over a 6-h treatment period. Overall, the CPTQ-treated group exhibited pronounced color changes compared to the negative control and the 7.5% HP group, while it showed comparable effects to those observed in the 30% HP group (Figure 5a). After 1.5 h of whitening, the color change (Δ*E*_00_) in the CPTQ group (6.22 ± 2.09) was already significantly superior to that in the control group (1.82 ± 0.56, *p* < 0.01, Figure 5b). And after 4.5 h of whitening, the color change (Δ*E*_00_) in the CPTQ group (10.94 ± 2.82) was already significantly superior to that in the 7.5% HP group (7.56 ± 1.78, *p* < 0.05, Figure 5b). During the 6 h bleaching procedure, significant differences between the CPTQ group and the 30% HP group were observed only at the 3 h time point (Figure 5b), indicating comparable whitening efficacy between CPTQ and 30% HP. The changes in lightness (ΔL*) followed a trend similar to that of Δ*E*_00_. At 1.5 h, the increase in lightness in the CPTQ group (6.00 ± 2.10) was significantly greater than that in the control group (1.00 ± 0.63, *p* < 0.01). Except at the 3 h time point, no significant differences in ΔL* were observed between the CPTQ group and the 30% HP group from 0.5 h to 6 h (*p* > 0.05) (Figure 5c). For the red–green coordinate, the a* value decreased during CPTQ treatment (Figure 5d), indicating a reduction in redness. Starting from 1.5 h, the reduction in the a* value was significantly greater in the CPTQ group (−2.83 ± 0.98) compared to that in the control group (−1.67 ± 0.75, *p* < 0.01), and no significant difference was detected between the CPTQ and 30% HP groups from 0.5 h to 6 h (*p* > 0.05, Figure 5d). Regarding the yellow–blue coordinate, the b* value decreased during CPTQ treatment (Figure 5e), indicating a reduction in the yellowness of the extracted teeth. After 3 h of treatment, the reduction in the b* value was significantly greater in the CPTQ group (−3.01 ± 1.55) compared to that in the control group (−0.67 ± 0.52, *p* < 0.05). No significant difference in Δb* was observed between the CPTQ and 30% HP groups from 0.5 h to 6 h (*p* > 0.05, Figure 5e).

### 3.5. The Role of ROS in CPTQ-Mediated Whitening

**Figure 6:**
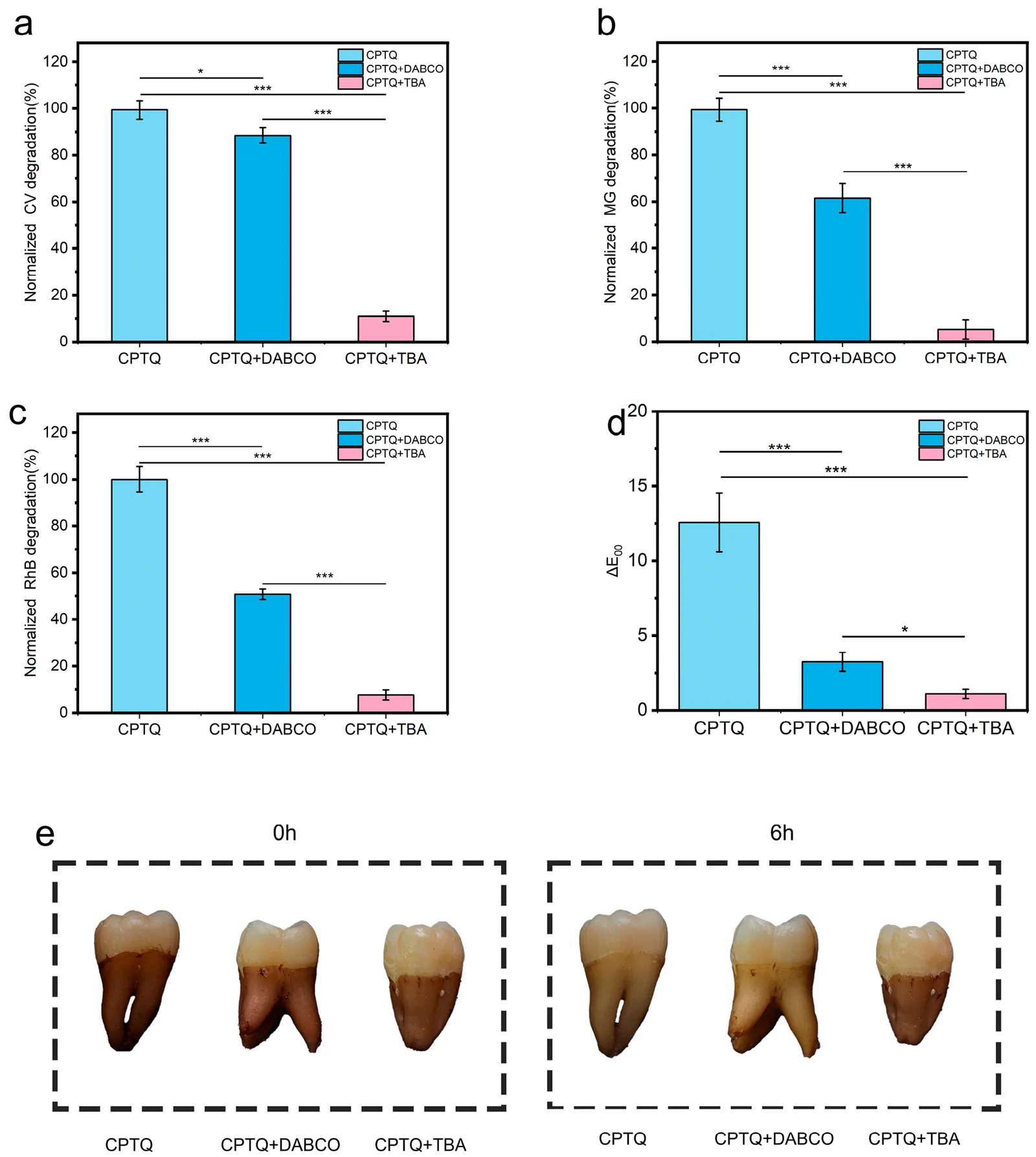
The role of Type I and Type II ROS in CPTQ-mediated pigment decomposition and tooth whitening. Normalized decomposition rates of CV (**a**), MG (**b**), and RhB (**c**) by CPTQ after the addition of DABCO and TBA. (**d**) Δ*E*_00_ of beverage-stained extracted teeth after CPTQ whitening with the addition of DABCO and TBA. (**e**) Photographs of extracted teeth from each group at the beginning and end of the whitening procedure. *** *p* < 0.001, * *p* < 0.05.

To investigate the relative contributions of Type I and Type II reactive oxygen species (ROS) in the CPTQ-mediated pigment degradation, tert-Butanol (TBA) and 1,4-Diazabicyclo[2.2.2]octane (DABCO) were used as scavengers of hydroxyl radicals (•OH) and singlet oxygen (^1^O_2_), respectively. The normalized decomposition rate was calculated to evaluate the contribution of each ROS type. In the CPTQ-mediated decomposition of CV, addition of DABCO moderately reduced the normalized decomposition rate to 88.46% ± 3.33% (*p* < 0.05), whereas TBA dramatically suppressed it to 10.89% ± 2.22% (*p* < 0.001) (Figure 6a). A similar trend was observed for MG, where the decomposition rate decreased to 61.43% ± 6.30% with DABCO (*p* < 0.001) and to 5.22% ± 4.08% with TBA (*p* < 0.001) (Figure 6b). For the RhB, DABCO reduced the normalized decomposition rate to 50.70% ± 2.23% (*p* < 0.001), whereas TBA further reduced it to 7.67% ± 2.17% (*p* < 0.001, Figure 6c). These results collectively indicated that hydroxyl radicals make a major contribution to pigment cleavage during CPTQ-mediated photo-whitening. To further evaluate whether these ROS contributed to tooth whitening, DABCO and TBA were applied to the CPTQ-based whitening system using beverage-stained extracted teeth. The color difference (Δ*E*_00_) was recorded and calculated. After 6 h of treatment, the *Δ*E_00_ in the control group was 12.56 ± 1.97, which decreased to 3.24 ± 0.63 upon addition of DABCO (*p* < 0.001) and dropped to 1.09 ± 0.31 upon addition of TBA (*p* < 0.001) (Figure 6d). Consistent with the quantitative analysis, photographic evaluation revealed evident color improvement in the CPTQ group and the CPTQ + DABCO group, while negligible change was observed in the CPTQ + TBA group (Figure 6e). These findings indicate that the tooth whitening effect mediated by CPTQ is primarily mediated by Type I ROS, particularly •OH.

### 3.6. Effects of CPTQ Whitening on Enamel Surface Properties

**Figure 7:**
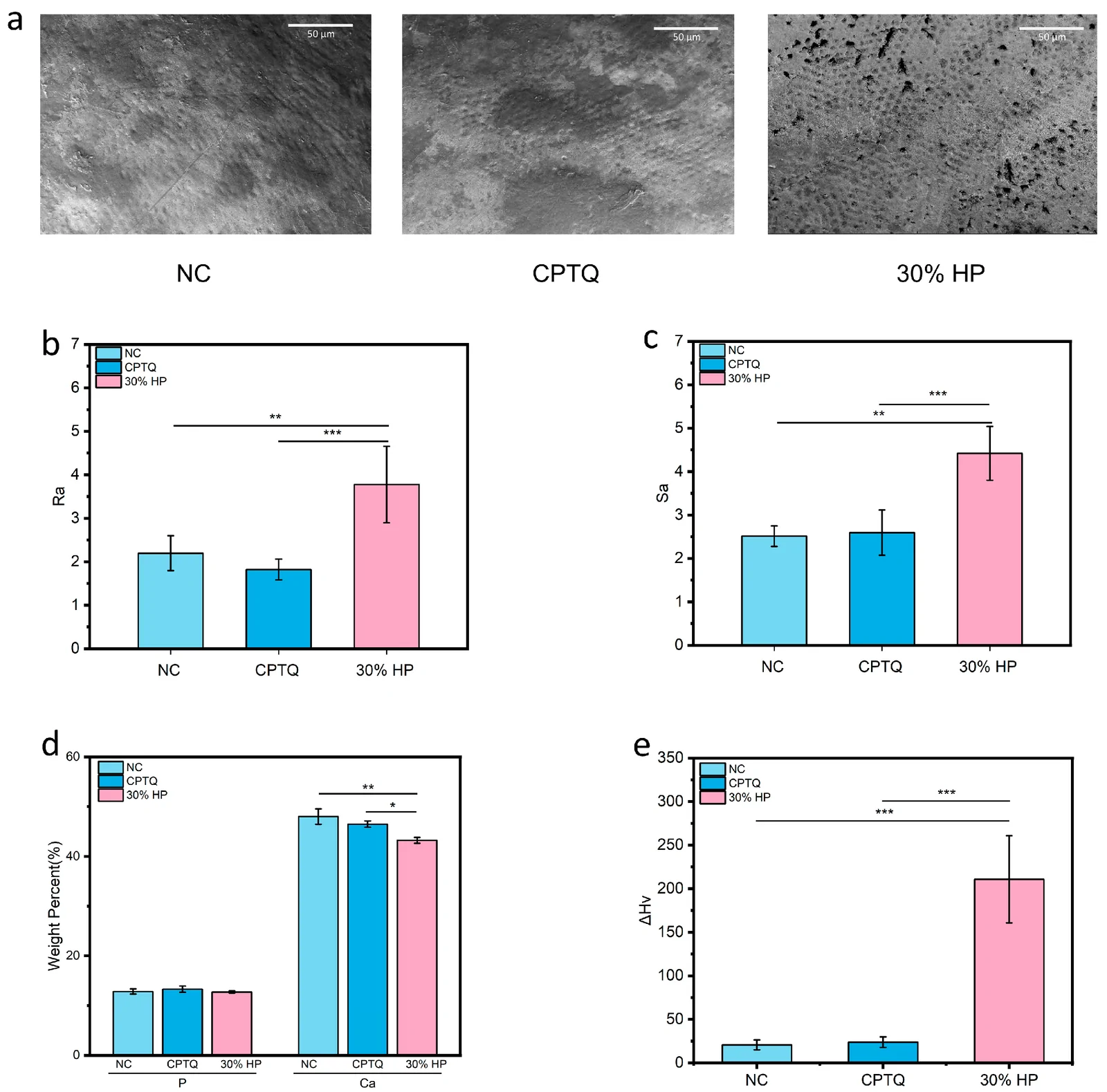
Surface topography characterization of enamel: (**a**) SEM images of enamel treated with NC, CPTQ, and 30% HP after the whitening procedure. (**b**,**c**) Characterization of *R*_a_ and *S*_a_. (**d**) EDS elemental analysis showing calcium and phosphorus content. (**e**) Comparison of enamel microhardness before and after the whitening procedure measured by a microhardness tester. *** *p* < 0.001, ** *p* < 0.01, * *p* < 0.05.

In the final step, a comprehensive evaluation of the morphological, chemical, and mechanical properties of the enamel was conducted. The morphology of the enamel surface was examined by scanning electron microscopy (SEM). Both the negative control group and the CPTQ-treated group exhibited regular and uniform enamel surface structures, indicating maintenance of surface integrity. In contrast, the 30% HP-treated group showed pronounced concave erosion of enamel prisms, accompanied by evident microcracks (Figure 7a). The roughness average (*R*_a_) and surface roughness average (*S*_a_) of the teeth were measured using a laser confocal microscope. No significant differences in *R*_a_ or *S*_a_ were observed between the CPTQ (*R*_a_ = 1.82 ± 0.24, *S*_a_ = 2.59 ± 0.52) and control groups (*R*_a_ = 2.19 ± 0.41, *S*_a_ = 2.51 ± 0.24, *p* > 0.05). However, 30% HP treatment significantly increased *R*_a_ (3.78 ± 0.88, *p* < 0.01) and *S*_a_ (4.22 ± 0.62, *p* < 0.01), indicating surface deterioration (Figure 7b,c). Energy-dispersive X-ray spectroscopy (EDS) was used to detect the content of calcium (Ca) and phosphorus (P) on the enamel surface. The results showed no significant changes in the content of Ca and P elements after CPTQ (Ca = 46.50% ± 0.62%, *p* = 13.30% ± 0.62%) treatment compared to the control group (Ca = 48.00% ± 1.54%, *p* = 12.83% ± 0.51%, *p* > 0.05). In contrast, the 30% HP group (Ca = 43.33% ± 0.57%) showed a significant reduction in Ca content compared with both the control group (*p* < 0.01) and CPTQ group (*p* < 0.05, Figure 7d), implying mineral loss. The hardness of the enamel was also measured after treatment. Consistently, enamel microhardness remained unchanged after CPTQ (Δ*H*_V_ = 23.80 ± 6.11, *p* > 0.05) treatment but decreased significantly following 30% HP treatment (Δ*H*_V_ = 210.74 ± 50.13, *p* < 0.001, Figure 7e).

## 4. Discussion

The aim of this study was to explore the application of the AIEgens photosensitizer developed by our group in dental whitening treatment. Currently, the widely accepted theory suggests that the tooth whitening process may primarily involve oxidative degradation of organic chromogenic macromolecules on or within the dental hard tissues [11,37]. The oxidative potential of ROS is closely associated with their pigment degradation capacity. For example, •OH has a higher oxidation potential than HP and may promote oxidative cleavage of chromophoric structures [23]. Consequently, CPTY and CPTQ, which were previously shown to generate Type I ROS, were considered potential photosensitizers for photodynamic tooth whitening. The results of the model pigment decomposition experiment indicate that both CPTY and CPTQ possess broad-spectrum pigment decomposition, which can be attributed to the highly oxidative hydroxyl radicals (•OH) primarily generated by these systems [38]. For the same pigment and concentration, CPTQ causes a greater reduction in pigment concentration and reaches the plateau phase earlier than CPTY, consistent with its stronger •OH generation capacity reported previously [32,33]. CPTY and CPTQ exhibited greater pigment decomposition than 7.5% HP and 30% HP under the present experimental conditions, particularly for RhB. This may be attributed to the fact that RhB is relatively resistant to oxidation and may require highly oxidative ROS, such as •OH, for effective decomposition, whereas hydrogen peroxide exhibits relatively weak oxidative decomposition effects on RhB [39,40].

The results described above demonstrated that CPTQ possesses greater model pigment decomposition compared to HP. To further explore the potential of CPTQ in tooth whitening, we evaluated its whitening efficacy on both model-stained and beverage-stained extracted teeth. The results showed that CPTQ produced significant color changes in both model pigment-stained and beverage-stained extracted teeth at early treatment time points. In addition to being significantly different from the control group, the Δ*E*_00_ after CPTQ treatment exceeded the commonly used perceptibility and acceptability thresholds in dental color studies [35], suggesting that the color change was visually perceptible and aesthetically meaningful [41]. Therefore, the color changes obtained in this study may be considered visually meaningful rather than merely statistically significant [42]. The 6 h irradiation period was used to characterize time-dependent whitening in this in vitro proof-of-concept study, rather than as a proposed clinical protocol. Prolonged treatment periods have also been reported in experimental tooth whitening studies [43,44]. Nevertheless, continuous 6 h irradiation would be impractical for conventional chairside treatment [45]. Our results show that the 1.5 h CPTQ treatment already exhibited a bleaching effect, which may suggest its potential for rapid whitening, but further experiments are still needed. For instance, we need to design protocols with shorter observation intervals and larger sample sizes to ensure detection sensitivity for short-term effects. Additionally, combining material science approaches will also help ensure sufficient photodynamic reactions, thereby further verifying the feasibility of applying CPTQ to in-office bleaching [46]. On the other hand, CPTQ showed a bleaching efficacy superior to 7.5% low-concentration HP throughout the 6 h continuous treatment. Considering its relatively low light power density (25 mW/cm^2^), CPTQ may be adapted for at-home whitening systems using wearable LED bleaching trays [47].

PDT exerts its therapeutic effects primarily through the generation of ROS. PDT-generated ROS arise mainly through Type I and Type II pathways, with •OH and ^1^O_2_ commonly used as representative ROS of these pathways, respectively [48]. In recent years, Type I ROS have attracted increasing attention in photodynamic research due to several mechanistic features compared to Type II pathways. With higher oxidation potentials among ROS, •OH can achieve efficient oxidative degradation of organic substrates [49,50]. Moreover, Type I reactions proceed mainly through electron transfer processes and are less dependent on environmental oxygen concentration, which may enhance their stability and applicability under diverse conditions [51]. In this study, TBA and DABCO were used as scavengers of •OH and ^1^O_2_, respectively. Compared with DABCO, the addition of TBA to the reaction system significantly inhibited the decomposition of the model pigment and the bleaching of the isolated teeth with staining. These findings indicate that •OH-related Type I pathways made an important contribution to CPTQ-mediated photodynamic whitening under the present experimental conditions. Unlike direct peroxide application, CPTQ-mediated PDT relies on light-activated ROS generation, which may allow better temporal control of the oxidative process [52]. These findings support further investigation of CPTQ-mediated PDT as a potential approach for tooth whitening. Given that CPTQ may form aggregates in aqueous media [53,54], its penetration behavior is expected to differ from that of small H_2_O_2_ molecules. Photodynamically generated ROS are highly reactive and short-lived and therefore act over limited diffusion distances near the photosensitizer [55]. Accordingly, CPTQ-mediated whitening may primarily affect extrinsic stains and superficial enamel chromophores. However, because CPTQ aggregation size and localization within enamel and dentin were not measured, its penetration depth and efficacy against deeper intrinsic dentinal stains remain uncertain.

Despite being the most prevalent clinical bleaching agent, the widespread application of HP is limited by a spectrum of adverse effects, such as tooth sensitivity and even severe pain, enamel demineralization, and chemical burns to the gingiva and surrounding soft tissues [36,56,57]. Previous studies have reported that PDT-assisted tooth bleaching may cause relatively limited alterations to dental hard tissues [28,44]. These findings are consistent with this study: following the CPTQ treatment, there were no significant alterations in surface roughness (*R*_a_ and *S*_a_), elemental composition (Ca and P), or Vickers microhardness (*H*_V_) of the enamel. Conversely, the increased roughness, reduced Ca content, and decreased hardness observed in the HP group collectively indicate adverse changes in enamel surface properties [58,59]. Previous studies have suggested that HP-induced enamel alterations may be related to oxidative degradation of the organic matrix, changes in mineralized structures, and the acidic environment of peroxide formulations [60]. In our study, CPTQ treatment was performed in a neutral environment, which may partly explain the limited enamel surface changes observed after treatment. Collectively, these findings suggest that CPTQ-mediated photodynamic whitening caused no detectable adverse changes in the evaluated enamel surface parameters under the present in vitro conditions. Previous studies from our group reported no obvious cytotoxicity of related AIEgens in human gingival epithelial cells and human gingival fibroblasts, together with low hemolytic activity [33], providing preliminary evidence for their biological compatibility.

The findings generally supported our hypothesis. CPTQ produced a greater tooth color change than the light-only vehicle control at selected time points and yielded more favorable outcomes for the evaluated enamel surface parameters than 30% HP. The ROS-scavenging results further supported a major contribution of •OH-related Type I pathways to CPTQ-mediated whitening under the present experimental conditions.

Further research and optimization are required to establish optimal irradiation parameters and treatment protocols. This study was limited to the direct comparison between CPTQ and HP. Incorporating commonly used carbamide peroxide (such as 10% CP) would provide a more comprehensive comparative analysis. Although a CPTQ-in-the-dark control was not included, the ROS-scavenging results support an important role for light-generated ROS, while light-independent CPTQ effects cannot be fully excluded. A normal light control group was lacking to rule out the potential activation of the molecule under ambient light. EDS was used to obtain semiquantitative information on the relative Ca and P signals on the enamel surface. However, EDS quantification, particularly for light elements, may be affected by X-ray absorption, surface topography, background interference, and matrix-correction procedures. Therefore, the present EDS results should be interpreted as relative elemental signals rather than absolute mineral contents. Future studies should employ standard-based EPMA-WDS for more rigorous quantitative analysis of Ca and P, with XPS used as a complementary surface-sensitive technique to characterize elemental composition and chemical states [61,62]. Notably, because surface roughness was assessed only after treatment, the *R*_a_ and *S*_a_ results represent post-treatment between-group comparisons rather than within-specimen changes from baseline. Additionally, investigations are needed to evaluate the whitening efficacy on teeth subjected to prolonged or more intense extrinsic staining encountered in actual clinical settings. In addition, the whitening effect and safety evaluation in this study were limited to in vitro experiments. Further studies, such as in vivo animal models and long-term cytotoxicity assays on pulp cells, are required. Within these limitations, the present findings indicate that Type I ROS–mediated photodynamic whitening may offer an alternative approach capable of balancing whitening efficacy with enamel preservation.

## 5. Conclusions

Our study demonstrates that CPTQ-mediated photodynamic tooth whitening achieves a whitening effect superior to 7.5% HP and comparable to that of 30% HP in both model pigment systems and stained extracted teeth. Mechanistic inhibition experiments indicate that the whitening efficacy may be primarily mediated by hydroxyl radical-driven Type I ROS, supporting a controllable oxidative mechanism distinct from conventional HP-based whitening. Importantly, CPTQ did not induce detectable alterations in enamel morphology, surface roughness, elemental composition, and microhardness, differing from the structural changes observed after 30% HP treatment. Thus, the hypothesis was partially supported: the predicted whitening advantage was observed at selected time points, and the predicted enamel surface outcomes were supported, although the whitening advantage was dependent on the staining model and treatment duration. These findings support further investigation of CPTQ as a peroxide-free photodynamic tooth whitening approach.

## Data Availability

The datasets during the current study are available from the corresponding author upon reasonable request.
